# Metastatic Type 1 low-grade gastric neuroendocrine tumor treated with peptide receptor radionuclide therapy in a young adult: a case report

**DOI:** 10.1093/gastro/goae023

**Published:** 2024-04-16

**Authors:** Elisabetta Dell’Unto, Maria Rinzivillo, Gianluca Esposito, Elsa Iannicelli, Daniela Prosperi, Francesco Panzuto, Bruno Annibale

**Affiliations:** Department of Medical-Surgical Sciences and Translational Medicine, Sapienza University of Rome, Rome, Italy; Digestive Disease Unit—ENETS Center of Excellence, Sant’Andrea University Hospital, Italy; Digestive Disease Unit—ENETS Center of Excellence, Sant’Andrea University Hospital, Italy; Department of Medical-Surgical Sciences and Translational Medicine, Sapienza University of Rome, Rome, Italy; Digestive Disease Unit—ENETS Center of Excellence, Sant’Andrea University Hospital, Italy; Department of Medical-Surgical Sciences and Translational Medicine, Sapienza University of Rome, Rome, Italy; Radiology Unit - ENETS Center of Excellence, Sant'Andrea University Hospital, Rome, Italy; Nuclear Medicine Unit—ENETS Center of Excellence, Sant’Andrea University Hospital, Rome, Italy; Department of Medical-Surgical Sciences and Translational Medicine, Sapienza University of Rome, Rome, Italy; Digestive Disease Unit—ENETS Center of Excellence, Sant’Andrea University Hospital, Italy; Department of Medical-Surgical Sciences and Translational Medicine, Sapienza University of Rome, Rome, Italy; Digestive Disease Unit—ENETS Center of Excellence, Sant’Andrea University Hospital, Italy

## Background

Gastric neuroendocrine tumors (g-NETs) are classified into: Type 1, associated with chronic atrophic gastritis (CAG); Type 2, related to Zollinger–Ellison Syndrome/Multiple Endocrine Neoplasia Type 1; and sporadic Type 3 [1]. Type 1, the most common, typically presents as small, well-differentiated tumors with excellent survival [1]. Tumor size and gender are important prognostic factors in Type 1 g-NETs, with a controversial prognostic role of the Ki-67 index [2, 3]. Metastatic Type 1 g-NETs are rare and treatment of advanced cases mirrors that of other advanced NETs, including somatostatin analogs (SSA) and peptide receptor radionuclide therapy (PRRT), though specific data are limited [1].

## Case presentation

**Figure 1.**
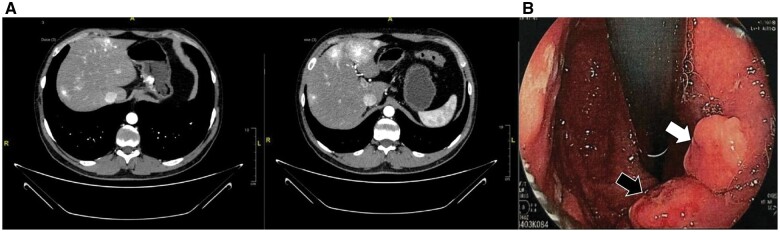
Radiological and endoscopic aspects of the g-NET. (A) Contrast-enhanced computer tomography with evidence of liver metastasis in both hepatic lobes and a hypervascular neoformation in the lesser curvature of the stomach. (B) Standard esophagogastroduodenoscopy (white-light) with the presence of two sessile lesions of 15 mm each—one covered by a normal mucosal pattern with normal vascularization (white arrow) and the other with a small sore at the center with an irregular vascular and mucosal pattern (black arrow).

In July 2020, a 34-year-old Caucasian man was referred to our center with a g-NET diagnosis, previously undetermined in type. Abdominal ultrasound, conducted due to dyspeptic symptoms, revealed liver metastasis; contrast-enhanced computed tomography (CT) further showed involvement in both hepatic lobes and a hypervascular gastric mass. Esophagogastroduodenoscopy (EGD) identified two sessile lesions in the gastric body (3 cm and 18 mm), confirmed as well-differentiated NET G1 (Ki67 < 2%, CgA+, Syn+, CD56+) (Figure 1). The patient began receiving lanreotide treatment from October 2020. At this time, the patient was asymptomatic.

In July 2021, he visited our center in good clinical condition. Blood tests showed macrocytosis (mean cell volume, 102 fL; reference range [RR], 80–96 fL), low vitamin B12 levels (<83 pg/mL [RR, 187–883 pg/mL]), and hyperhomocysteinemia (46.2 µmol/L [RR, 4–13.5 µmol/L]), despite no anemia (hemoglobin, 16.8 g/dL [RR, 13–17 g/dL]). Elevated chromogranin A and gastrin levels were noted (340.4 ng/mL [R.R. 0–100 ng/mL] and 515 pg/mL [R.R. 25–111 pg/mL], respectively). Tests indicated elevated antiparietal cell antibody at 54.36 IU/mL (RR, 0–30 IU/mL) and reduced pepsinogen I at 18 μg/L (RR, 30–120 μg/L). Additionally, the patient had an elevated thyroid peroxidase antibody level (53 IU/mL [R.R. <35 IU/mL]) and a high thyroid stimulating hormone level (4.8 mU/L [RR, 0.15–3.5 mU/L]), but normal FT-4 level (20 pmol/L [RR, 10–25]). EGD with biopsies from the gastric antrum and fundus/body showed CAG, sparing the gastric antrum, with no *Helicobacter pylori* infection detected. Based on these findings, the gastric lesion was classified as a CAG-related Type 1 g-NET.

Disease staging was completed by using 68Ga-DOTATOC positron emission tomography-CT and chest CT scans, showing high somatostatin receptor (SST) expression. The patient remained stable under lanreotide until October 2021 when a CT showed increased liver metastasis size (62 mm × 40 mm). Given the high SST expression and the evidence of radiological progression under lanreotide, he underwent four cycles of PRRT (800 mCi of Lu177-oxodotreotide) between November 2021 and May 2022. During the treatment, the patient remained asymptomatic and experienced no deterioration in quality of life. Three months later, CT showed stable disease with reduced lymphadenopathy. Repeat EGD revealed smaller gastric lesions (15 mm each), one with normal mucosa and the other with irregular mucosal and vascular patterns. Biopsies confirmed one lesion as NET G1 (Ki67 1%) and the other as metaplastic CAG with low-grade intestinal epithelial dysplasia. After these findings, the lesions were successfully removed via endoscopic mucosal resection. Histological analysis of the second lesion revealed a coexisting tubular adenoma with low-grade intestinal dysplasia and a NET G1 (Ki67 2%, well-differentiated tumor cell nets surrounded by fibrous stroma, chromogranin and synaptophysin positive staining, no mitosis), both extending to the deep resection margin (R1) and measuring 14 mm in size. Although an R1 finding was present, the patient underwent surveillance without further endoscopic enlargement. A new liver biopsy on metastatic lesions reaffirmed the diagnosis of a NET G1 (Ki-67 < 1%). The patient maintained stable disease under ongoing lanreotide treatment, as confirmed by using regular CT and 68Ga-DOTATOC PET-CT scans until the last follow-up in June 2023.

## Discussion

We report a rare case of metastatic Type 1 G1 g-NET. Type 1 g-NETs are typically treated conservatively using endoscopy, reflecting their benign nature and good prognosis, often leading to prolonged survival [1, 2]. Although they have a high recurrence rate, their malignancy risk is low and dependent on tumor size [3]. Endoscopic management is both safe and effective, ensuring high survival and enabling early detection of recurrences [1].

This case report is noteworthy for several reasons, as follows:

- The case involves a young patient with substantial hepatic disease, uncommon in Type 1 g-NETs, which are typically seen in older patients. In a large series, only 8 out of 20 patients with metastatic Type 1 g-NET had liver metastases, with a median age of 66 years [4]. Borch *et al.* [5] reported a single case among 65 patients with a median age of 55.1 years and Spampatti *et al.* [6] described only a 60-year-old patient with metastatic Type 1 g-NET, progressing from a well-differentiated to a high-grade G3 tumor.
- The histological finding of a tubular adenoma with low-grade intestinal dysplasia alongside a NET, post-endoscopic resection is intriguing. While CAG predisposes patients to intestinal-type gastric cancer, with dysplasia being a precursor, the concurrent presence of neuroendocrine and non-neuroendocrine lesions in the same patient is exceedingly rare. Our findings align with those of literature, showing CAG as a risk factor for both stomach adenocarcinoma and NET [7]. Additionally, the patient’s CAG, accompanied by pernicious anemia and autoimmune thyroid disease, is linked to an increased relative risk of gastric adenocarcinoma [8].
- Our literature review on therapeutic strategies for metastatic Type 1 g-NETs revealed no cases treated with PRRT. Although the effectiveness of PRRT in advanced entero-pancreatic NETs is established [9, 10], data for gastric-origin NETs are scarce. According to the European Neuroendocrine Tumor Society recommendations, PRRT could be a valid strategy in case of a high SST expression; everolimus could also be considered, especially in cases with low SST expression [1]. To our knowledge, no further information about this clinical context is available in the literature.

In summary, this case involves a young man diagnosed with metastatic Type 1 g-NET, characterized by a significant disease burden at diagnosis despite a low proliferative index. Initially aggressive, the disease showed a favorable response to PRRT and subsequently stabilized. This case also exhibited concurrent neuroendocrine and non-neuroendocrine lesions, underscoring the potential of CAG to develop into NET and adenocarcinoma.

## Authors’ Contributions

Conceptualization: B.A. and F.P. Original draft preparation: M.R. and E.D. Reviewing and editing: G.E., F.P., and B.A. All authors read and approved the final manuscript.

## References

[1] Panzuto F , Ramage J, Pritchard DM et al European Neuroendocrine Tumor Society (ENETS) 2023 guidance paper for gastroduodenal NET G1-G3. J Neuroendocrinol 2023;35:e13306. 37401795 10.1111/jne.13306

[2] Panzuto F , Parodi MC, Esposito G et al Endoscopic management of gastric, duodenal and rectal NETs: Position paper from the Italian Association for Neuroendocrine Tumors (Itanet), Italian Society of Gastroenterology (SIGE), Italian Society of Digestive Endoscopy (SIED). *Dig Liver Dis* 2024;56:589–600. 10.1016/j.dld.2023.12.015 38216439 38216439

[3] Panzuto F , Campana D, Massironi S et al Tumour type and size are prognostic factors in gastric neuroendocrine neoplasia: a multicentre retrospective study. Dig Liver Dis 2019;51:1456–60. 31175013 10.1016/j.dld.2019.04.016

[4] Grozinsky-Glasberg S , Thomas D, Strosberg JR et al Metastatic type 1 gastric carcinoid: a real threat or just a myth? World J Gastroenterol 2013;19:8687–95. 24379587 10.3748/wjg.v19.i46.8687 PMC3870515

[5] Borch K , Ahrén B, Ahlman H et al Gastric carcinoids: biologic behavior and prognosis after differentiated treatment in relation to type. Ann Surg 2005;242:64–73. 15973103 10.1097/01.sla.0000167862.52309.7d PMC1357706

[6] Spampatti MP , Massironi S, Rossi RE et al Unusually aggressive type 1 gastric carcinoid: a case report with a review of the literature. Eur J Gastroenterol Hepatol 2012;24:589–93. 22465973 10.1097/MEG.0b013e328350fae8

[7] Lahner E , Esposito G, Pilozzi E et al Gastric cancer in patients with type I gastric carcinoids. Gastric Cancer 2015;18:564–70. 24890255 10.1007/s10120-014-0393-8

[8] Vannella L , Lahner E, Osborn J et al Systematic review: gastric cancer incidence in pernicious anaemia. Aliment Pharmacol Ther 2013;37:375–82. 23216458 10.1111/apt.12177

[9] Strosberg JR , Caplin ME, Kunz PL et al; NETTER-1 Investigators. 177Lu-Dotatate plus long-acting octreotide versus high-dose long-acting octreotide in patients with midgut neuroendocrine tumours (NETTER-1): final overall survival and long-term safety results from an open-label, randomised, controlled, phase 3 trial. Lancet Oncol 2021;22:1752–63. 34793718 10.1016/S1470-2045(21)00572-6

[10] Brabander T , van der Zwan WA, Teunissen JJM et al Long-term efficacy, survival, and safety of [^177^Lu-DOTA^0^,Tyr^3^]octreotate in patients with gastroenteropancreatic and bronchial neuroendocrine tumors. Clin Cancer Res 2017;23:4617–24. 28428192 10.1158/1078-0432.CCR-16-2743

